# Circular RNA WHSC1 exerts oncogenic properties by regulating miR‐7/TAB2 in lung cancer

**DOI:** 10.1111/jcmm.16925

**Published:** 2021-09-22

**Authors:** Sisi Guan, Li Li, Wen‐Shu Chen, Wen‐Yang Jiang, Yun Ding, Li‐Lan Zhao, Yi‐Fan Shi, Jun Wang, Qi Gui, Cheng‐Cheng Xu, Yang Cheng, Wenjuan Zhang

**Affiliations:** ^1^ Department of Geriatrics The Central Hospital of Wuhan Tongji Medical College Huazhong University of Science and Technology Wuhan China; ^2^ Department of Thoracic Surgery Shengli Clinical Medical College of Fujian Medical University Fuzhou China; ^3^ Department of Thoracic Surgery Renmin Hospital of Wuhan University Wuhan China; ^4^ Department of Thoracic Surgery The First Affiliated Hospital of Soochow University Suzhou China; ^5^ Department of Oncology The First Affiliated Hospital of Soochow University Suzhou China

**Keywords:** circ‐WHSC1, miR‐7, non‐small cell lung cancer, TAB2

## Abstract

Circular RNA is a newly discovered member of non‐coding RNA (ncRNA) and regulates the target gene by acting as a micro‐RNA sponge. It plays vital roles in various diseases. However, the functions of circular RNA in non‐small cell lung cancer (NSCLC) remain still unclear. Our data showed that circ‐WHSC1 was highly expressed in NSCLC cells and tissues. Both in vitro and in vivo experiments showed that circ‐WHSC1 promoted NSCLC proliferation. circ‐WHSC1 also promoted the migration and invasion of lung cancer cells. Through bioinformatic analysis and functional experiments, we showed that circ‐WHSC1 could act as a sponge for micro‐RNA‐7 (miR‐7) and regulate the expression of TAB2 (TGF‐beta activated kinase one binding protein two). Inhibition of the circ‐WHSC1/miR‐7/TAB2 pathway could effectively attenuate lung cancer progression. In summary, this study confirmed the existence and oncogenic function of circ‐WHSC1 in NSCLC. The research suggests that the circ‐WHSC1/miR‐7/TAB2 axis might be a potential target for NSCLC therapy.

## 1 INTRODUCTION

Lung cancer is a cancer with high malignancy.^1^ Lung cancers can be classified as small cell lung cancer (SCLC) and non‐small cell lung cancer (NCSLC). NSCLC accounts for 85% of lung cancers.^2^

Despite the development of diagnosis and treatments for lung cancer, the survival of lung cancer remains very poor. Conventional chemotherapy and surgery showed limited effects, especially for NSCLC patients in metastatic stages.^3^

There is a lack of early diagnosis methods and effective therapies for lung cancer that exhibits high growth rates and extensive metastasis.^4^ The elucidation of the lung cancer progression would provide more potential targets for personalized therapeutic strategies.

Recent researches show that non‐coding RNAs (ncRNAs) are of great importance in various biological processes. ncRNAs are groups of RNA that could not encode any proteins, and micro‐RNAs and lncRNAs are the most investigated ncRNAs. Circular RNAs (circRNAs) are newly discovered ncRNAs that are widely expressed in different cells. It has been widely known that miRNAs and lncRNAs play critical roles in the regulation of disease progression.^5^, ^6^ However, the functions of circular RNAs require further investigation.

circRNAs are closed loops with 5′caps and 3′ covalently connected. circRNAs are transcribed via non‐canonical splicing and are resistant to RNase digestion.^7^ With the development of bioinformatic analysis and sequencing technologies, more and more circRNAs have been identified.^8^, ^9^ Research suggests that an abnormal expression of circRNA contributes to the development of various cancers, such as lung cancer,^10^, ^11^ hepatocellular cancer^12^ and colorectal cancer.^13^ The discovery of circular RNA in lung cancers and in‐depth researches on the specific molecular mechanisms may improve the therapy of lung cancer.

We carried out biology experiments to investigate the function of circRNAs in lung cancer in this research.^14^ From the total RNA, 5,471 distinct circRNAs have been identified. And more than 180 circRNAs were shown to have different expression levels between adjacent normal and cancerous tissues. A circular RNA transcribed from the exon of the *WHSC1* gene (Wolf‐Hirschhorn Syndrome Candidate 1), termed circ‐WHSC1 (hsa_circ_0004156), was observed to be increased in NSCLC tissues (fold change: 19.7).^14^ Our study aims to investigate the potential carcinogenic mechanism of circ‐WHSC1 in NSCLC.

## 2 METHODS

### 2.1 Cell culture

A549, NCI‐H460, HCC827, NCI‐H292, 95‐D, NCI‐H358, SW1537 and BEAS‐2B were from ATCC (The Global Bioresource Center). BEAS‐2B was cultured in LONZA BEBM Basal Medium kit. A549 and H460 cells were cultured in DMEM containing 10% FBS. Other cells were cultured in RPMI1640 medium with 10% FBS. All cells were maintained with 100 U/ml penicillin and100 mg/ml streptomycin at 37°C.

### 2.2 Patient samples

We collected fifteen paired NSCLC tumour tissues and adjacent normal tissues from The Central Hospital of Wuhan. All samples were stored at −80°C until used. All tissues were collected under the direction of The Central Hospital of Wuhan. Each patient had been informed and consented to the use of tissues.

### 2.3 qRT‐PCR

We employed the RNA Extraction Kit (Invitrogen) to isolate RNA from tissue samples and lung cancer cell lines under the manufacture's instructions as previously described.^15^ To remove the influence of the linear RNA, we treated the total RNA with 2 U/μg RNaseR (Epicentre Technologies, RNR07250) for 60 min at 37.0°C. PCR of mRNA and circRNA was performed by RT Reagent Kit (TaKaRa). micro‐RNA RT PCR reaction was performed out by PowerSYBR Green PCR kit (ABI). The expressions of genes were normalized to GAPDH through ΔΔCt method.

### 2.4 Western blot

We separated the protein by SDS‐PAGE. The proteins were identified by chemiluminescence kit (Invitrogen). Specific Western blot methods could be found in previous research.^2^

### 2.5 Cell proliferation assay

Cell proliferation was detected by CCK8 Kit from Dojindo Molecular Technologies (DJDB4000X). We seeded 5000 lung cancer cells per well. At indicated time, 10 µl reagent was added to the well. Two hours later, we applied Synergy H4 (BioTek) to identify the absorbance at 450 nm.

### 2.6 Plasmids and transfection

Plasmids were constructed as described in previous study.^15^ The sequence of circ‐WHSC1 was cloned into pcDNA3.1 vector by Geneseed. The plasmids were transfected into cells by Lippo3000 according to instructions of manufactory (Invitrogen).

### 2.7 Wound healing assay

Cells were cultured overnight to achieve 100% density. We made the scratches by pipettes, and then washed the dishes with phosphate buffer solution. We treated the lung cancer cells with 10 μg/ml mitomycin C 5 h before the scratches. The cells were cultured in basic media without FBS for 24 h. Photographs were taken by microscopy immediately at 0 h and 24 h.

### 2.8 Cell invasion assays

Invasion assays were carried out using transwell chamber with Matrigel (Invitrogen) following the guideline of the manufactory. After incubating the cells for 48 h, we fixed the cells with 4% PFA. Then, we stained the cells with crystal violet.

### 2.9 Xenografts in nude mice

We obtained 4 to 6‐week old nude mice from SLAC Laboratory Animal (Shanghai, China). 3 × 10^6^ HCC827 cells were inoculated into the nude mice subcutaneously. Volumes of tumours were monitored every 3 days following the formula: 1/2 (length × width^2^). Tumours were collected and photographed 48 days after transplantation. We recorded the volume and weight of the tumour. All experiments were under the directions of The Central Hospital of Wuhan Animal Care and Use Committee.

### 2.10 Luciferase reporter assay

The sequence of miR‐7 or TAB2 3′UTR was inserted into pGL3 plasmids. It was constructed by GeneGreat Company. We used Lipofectamine 3000 to transfect pGL3‐Luciferase and pGL3‐Renal into lung cancer cells. The Luciferase and Renilla activities were detected 48 h later. Relative Luciferase activities were normalized to Renilla activities.

### 2.11 RNA pull‐down assay

We used biotin to label wide‐type or miR‐7‐binding site mutant cir‐WHSC1, and then conjugated to beads. Then, we incubated the beads in cell lysis with shaking overnight at 4°C. Then, we washed down the beads and eluted RNA. The experiments were also performed with wild type or mutant TAB2‐labelled with biotin.

### 2.12 Statistical analysis

All results were calculated and analysed by GraphPad Prism 6. The results were shown as mean ± SD. The significance was calculated by one‐way analysis of variance (ANOVA) or Student's *t*‐test.^16^ *p* < 0.05 was identified as significant.

## 3 RESULTS

### 3.1 Circular RNA WHSC1 is upregulated in NSCLC

**FIGURE 1:**
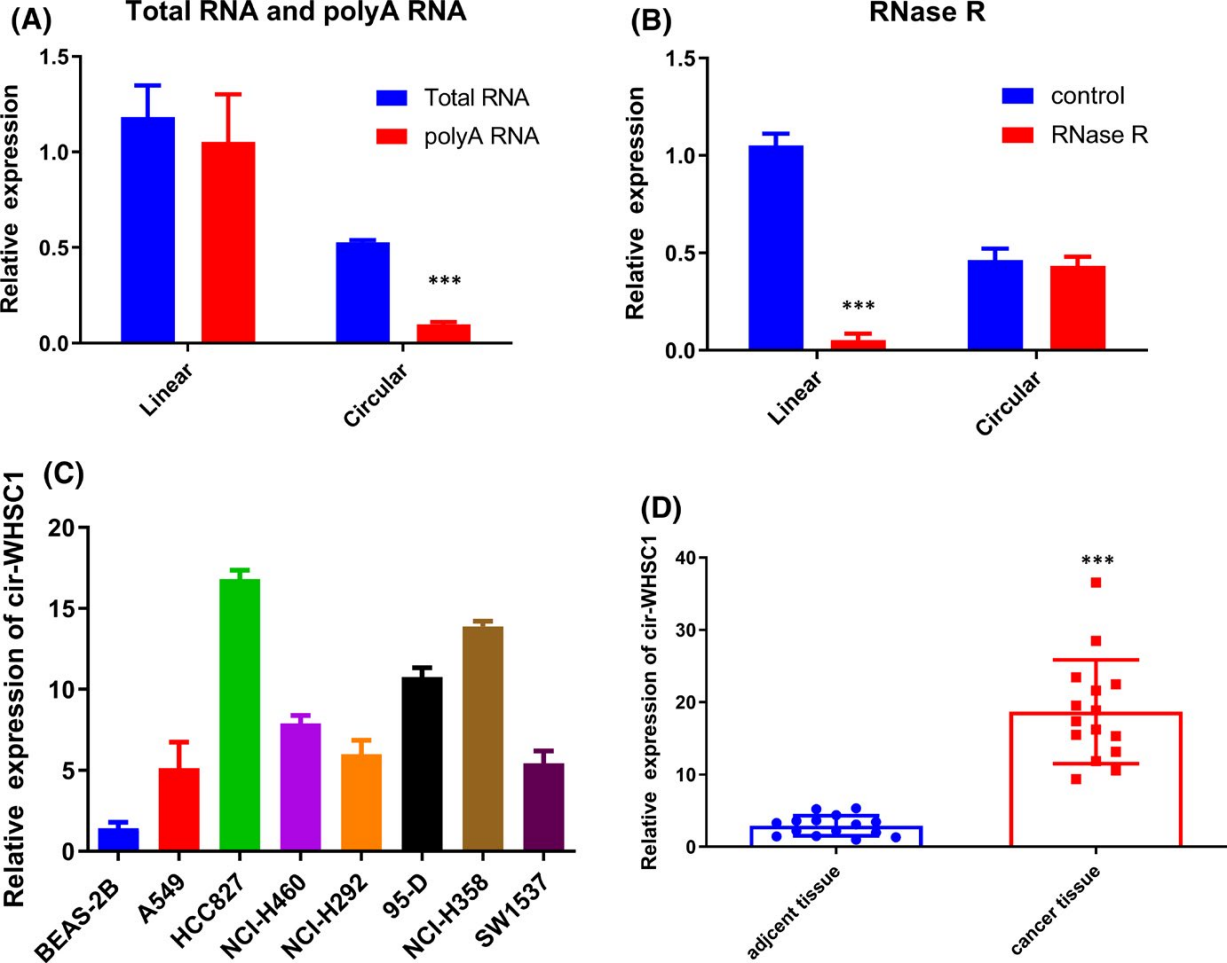
Detection of circ‐WHSC1 in NSCLC. (A) Random primers or oligodT was used in qRT‐PCR experiments in HCC827 cell line. The linear mRNA of WHSC1 could be amplified in both total RNA and poly (A) enriched RNA, while circ_WHSC1 level significantly decreased in poly (A) enriched RNAs. (B) We pretreated RNA with RNase R and then performed qRT‐PCR. circ‐WHSC1 was resistant to the digestion of RNase R. (C) Detection of circ‐WHSC1 in NSCLC cell lines. (D) Detection of circ‐WHSC1 in NSCLC tissues and adjacent tissues. NSCLC, non‐small cell lung cancer

A previous study showed that 180 circular RNAs had different expression levels in lung cancer tissue.^14^ We carried out experiments to confirm the presence of circ‐WHSC1 in NSCLC cells. Divergent and convergent primers were specially designed to amplify circ‐WHSC1 and linear WHSC1 exons. We performed qRT‐PCR using cDNA and genomic DNA. The qRT‐PCR data exhibited that the convergent primers could amplify the linear WHSC1 exon in both gDNA and cDNA, while divergent primers could only amplify the circular WHSC1 in cDNA (Figure 1A). Circular RNA is more resistant to RNase R digestion than its linear isoform because of the stability of its circular structure. To confirm this, RNA was pretreated with or without RNase R before the amplification of circ‐WHSC1. The results showed that RNase R treatment greatly decreased the level of linear WHSC1 expression without affecting the circ‐WHSC1 level (Figure 1B). Moreover, circ‐WHSC1 exhibited higher expressions in NSCLC cell lines than normal lung epithelial BEAS‐2B cells (Figure 1C). HCC827 and NCI‐H358 cells had relatively higher expressions of circ‐WHSC1 than A549 and NCI‐H292 cells. We selected HCC827, NCI ‐H292, NCI‐H358 and A549 cell lines for further experiments. Moreover, circ‐WHSC1 was also shown to display higher expressions in NSCLC tissues compared with that in the adjacent normal tissues (Figure 1D).

### 3.2 circ‐WHSC1 does not influence WHSC1 protein level

**FIGURE 2:**
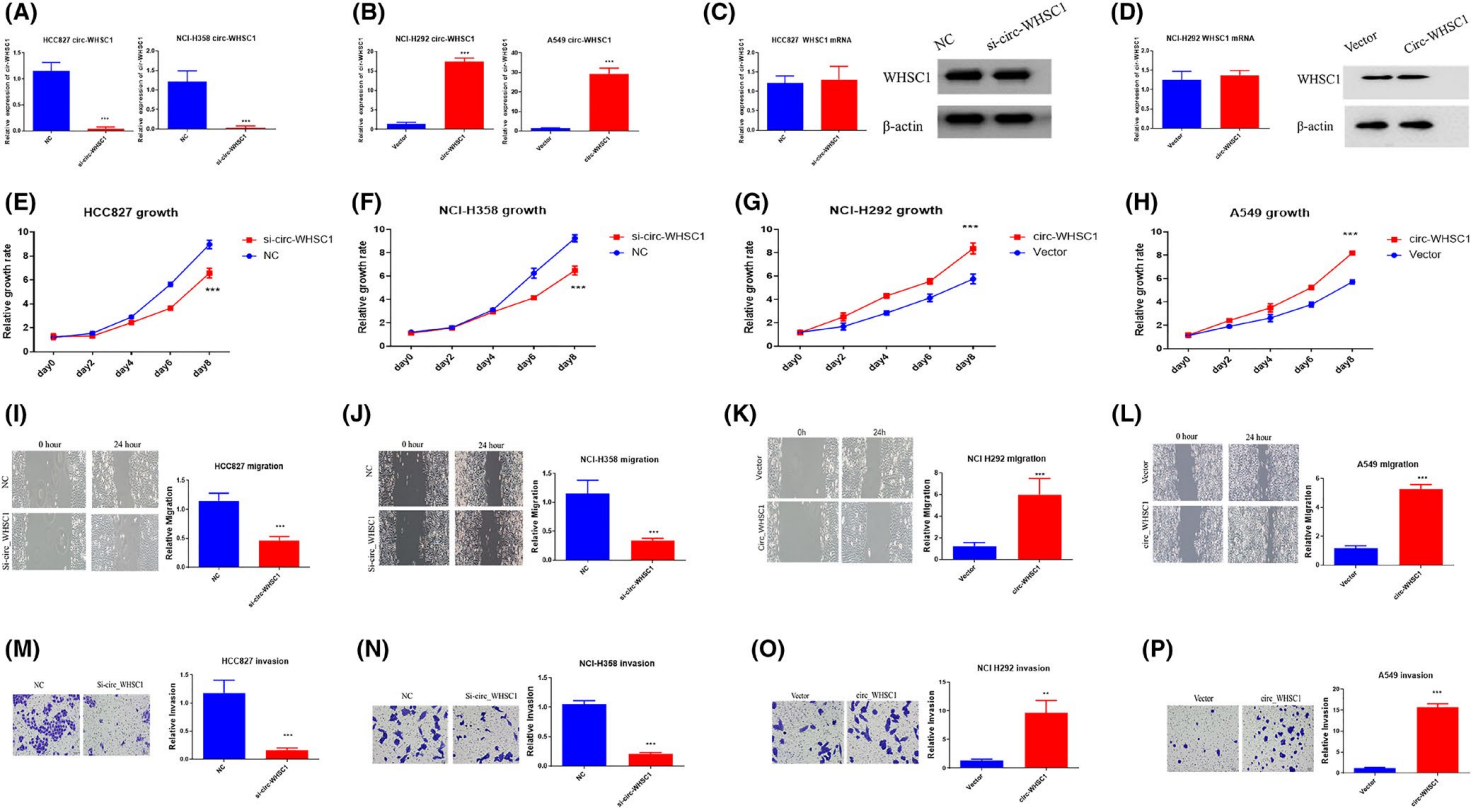
circ‐WHSC1 promotes the proliferation, migration and invasion of NSCLC cells without influencing WHSC1 expression. (A‐B) We transfected nonsense siRNA and circ‐WHSC1 siRNA into HCC827 and NCI‐H358 cells, and performed qRT‐PCR to detect circ‐WHSC1 level. (B) We transfected vector and circ‐WHSC1 overexpression plasmids into NCI‐H292 and A549 cells, and performed qRT‐PCR to detect circ‐WHSC1 level. (C) WHSC1 mRNA and protein level in HCC827. (D) WHSC1 mRNA and protein level in NCI‐H292. (E‐H) The tumour growth rates were detected in indicated NSCLC cell lines by CCK8 assays. (I‐L) The migration abilities were identified in indicated NSCLC cell lines by Wound Healing Assays. (M‐P) The invasion abilities were identified in indicated NSCLC cell lines by Invasion Assay. NSCLC, non‐small cell lung cancer

It is reported that circular RNA could affect the splicing of pre‐RNA, which in turn influences the mRNA and protein levels of the gene.^17^ First, we performed experiments to determine whether circ‐WHSC1 could affect WHSC1 mRNA and protein levels. siRNAs targeting circ‐WHSC1 were transfected into HCC827 and NCI‐H358 cells. As indicated, siRNAs could effectively reduce the level of circ‐WHSC1 (Figure 2A). Then, we detected WHSC1 mRNA and protein levels. The results showed that mRNA and protein expression of WHSC1 were not influenced by the silence of circ‐WHSC1 (Figure 2C). We also transfected circ‐WHSC1 overexpression plasmids into NCI‐H292 and A549 cell lines (Figure 2B). Similar outcomes were obtained from NCI‐H292 cells, in which the circ‐WHSC1 did not regulate the WHSC1 expression (Figure 2D). In conclusion, our results confirmed that circ‐WHSC1 could not influence WHSC1 mRNA and protein levels.

### 3.3 circ‐WHSC1 exerts oncogenic properties in NSCLC

We further investigated the biological roles of circ‐WHSC1 in NSCLC. We first detected the influence of circ‐WHSC1 on lung cancer cell growth. CCK8 was applied to identify the growth rate in HCC827, NCI‐H358, NCI‐H292 and A549 cells. The data exhibited that silencing of circ‐WHSC1 significantly weakened cancer cell proliferation (Figure 2E,F), and overexpression of circ‐WHSC1 increased proliferation (Figure 2G,H).

The functions of circ‐WHSC1 in migration and invasion were detected. The data proved that silencing of circ‐WHSC1 weakened the migration ability of HCC827 and NCI‐H358 cells significantly (Figure 2I,J), and the overexpression of circ‐WHSC1 increased migration in NCI‐H292 and A549 (Figure 2K,L). Invasion assay showed that circ‐WHSC1 contributed to the invasion of lung cancer cells (Figure 2M‐P). Thus, these data collectively suggest that circ‐WHSC1 plays important roles in promoting proliferation and mobility of lung cancer cells.

### 3.4 circ‐WHSC1 acts as a sponge for miR‐7

It is well known that circRNAs could function as sponges for micro‐RNAs and regulate their levels.^18^ We searched for the possible targeted micro‐RNA of circ‐WHSC1 using Circinteractome circular RNA database. The database predicted that miR‐7 had a binding site on circ‐WHSC1 and may be a potential target of circ‐WHSC1. The predicted binding site between circ‐WHSC1 and miR‐7 is listed in Figure S1.

**FIGURE 3:**
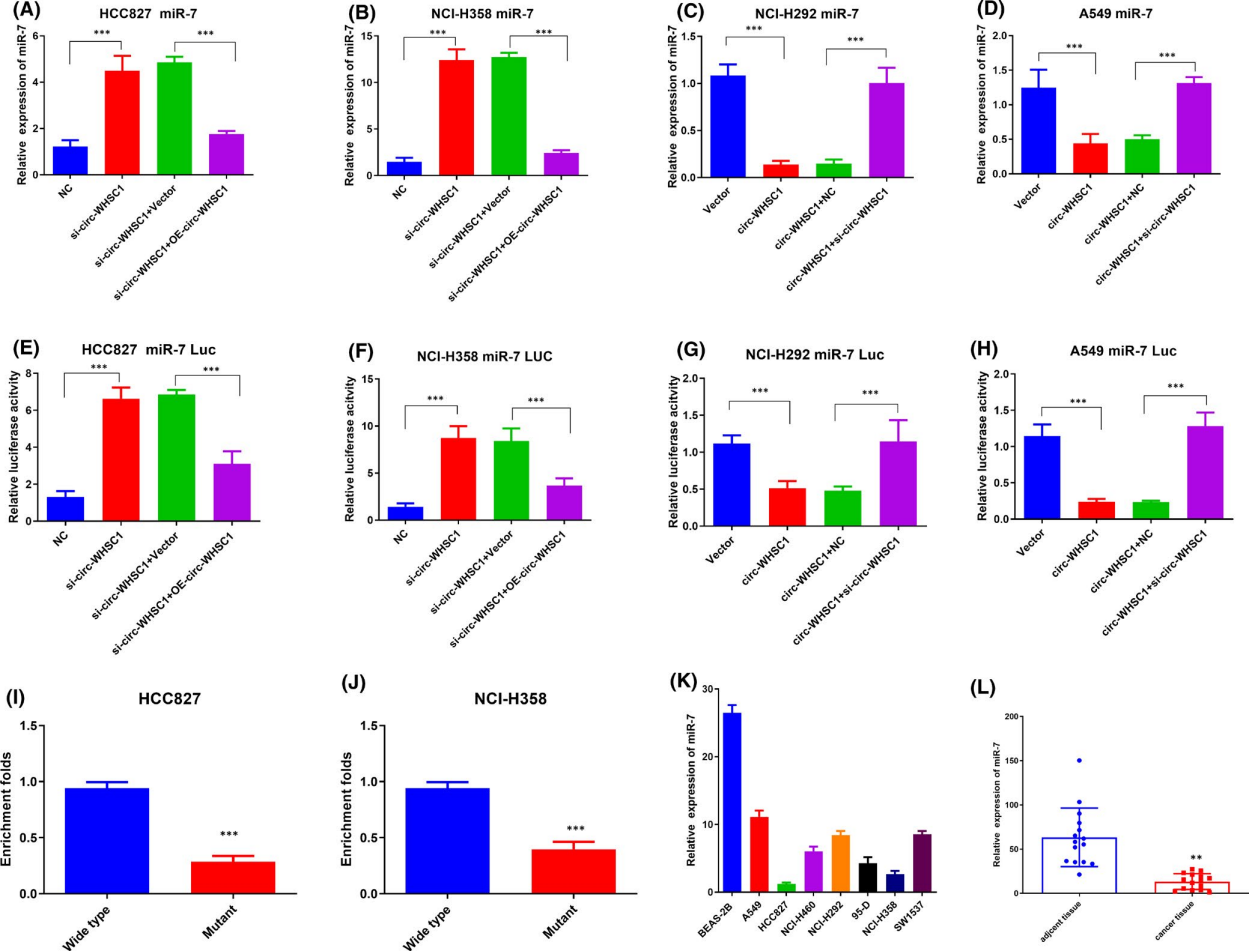
circ‐WHSC1 regulates miR‐7 level by acting as a sponge. (A) We silenced circ‐WHSC1 and then re‐expressed circ‐WHSC1 in HCC827 cells. miR‐7 level was detected using qRT‐PCR. (B) We silenced circ‐WHSC1 and then re‐expressed circ‐WHSC1 in NCI‐H358 cells. (C) We overexpressed the circ‐WHSC1 and then silenced circ‐WHSC1 in NCI‐H292 cells. miR‐7 level was detected using qRT‐PCR. (D) We overexpressed the circ‐WHSC1 and then silenced circ‐WHSC1 in A549 cells. miR‐7 level was detected using qRT‐PCR. (E‐H) Luciferase assays were performed in indicated lung cancer cells. (I‐J) Pull down of miR‐7 by wildtype or binding site‐mutated circ_WHSC1 from HCC827 (I) and NCI‐H358 (J) cell lysis. (K) miR‐7 levels were detected in NSCLC cell lines. (L) miR‐7 levels were detected in NSCLC tissues. NSCLC, non‐small cell lung cancer

To determine the relationship between circ‐WHSC1 and miR‐7, qRT‐PCR, luciferase assays and RNA pull‐down assays were carried out in lung cancer cells. First, we detected the miR‐7 level using qRT‐PCR after overexpressing or silencing circ‐WHSC1. Data proved that the silencing of circ‐WHSC2 upregulated miR‐7 level in HCC827 and NC1‐H358 (Figure 3A,B), while the overexpression of circ‐WHSC1 significantly decreased miR‐7 level (Figure 3C,D). Furthermore, the rescue experiments could effectively recover the level of miR‐7 indicating the specific regulation of miR‐7 by circ‐WHSC1 (Figure 3A‐D). In addition, we transfected luciferase plasmids containing miR‐7 sequence into cells to perform the luciferase assay. High level of circ‐WHSC1 significantly inhibited the luciferase activity, and the silencing of circ‐WHSC1 enhanced the luciferase activity (Figure 3E‐H). Rescue experiments also effectively restored the luciferase activity of miR‐7 (Figure 3E‐H).

To further investigate the interaction between circ‐WHSC1 and miR‐7, we performed RNA pull‐down experiment in NSCLC cell lines. Wild‐type and miR‐7‐binding site‐mutated circ‐WHSC1 labelled with biotin were used for the experiments. As indicated, the binding site mutation almost completely abolished the interaction between circ‐WHSC1 and miR‐7 in HCC827 and NCI‐H358 cells (Figure 3I,J). We also detected the miR‐7 level in NSCLC cell lines and tissues. The data showed that miR‐7 was highly expressed in normal cells and adjacent tissues, which had lower expressions of circ‐WHSC1 (Figure 3K,L). All these data collectively confirm that circ‐WHSC1 regulates miR‐7 by functioning as a micro‐RNA sponge.

### 3.5 TAB2 is the target gene of miR‐7

One of the main functions of miRNA is to reduce the stabilities of mRNA and then influence the transcription.^19^ TargetScan was applied to search for the targets of micro‐RNA‐7. TargetScan predicted that TAB2 might be the potential target of miR‐7. TAB2 could activate MAP3K7/TAK1 pathway and plays critical roles in various biological pathways.^20^, ^21^, ^22^

**FIGURE 4:**
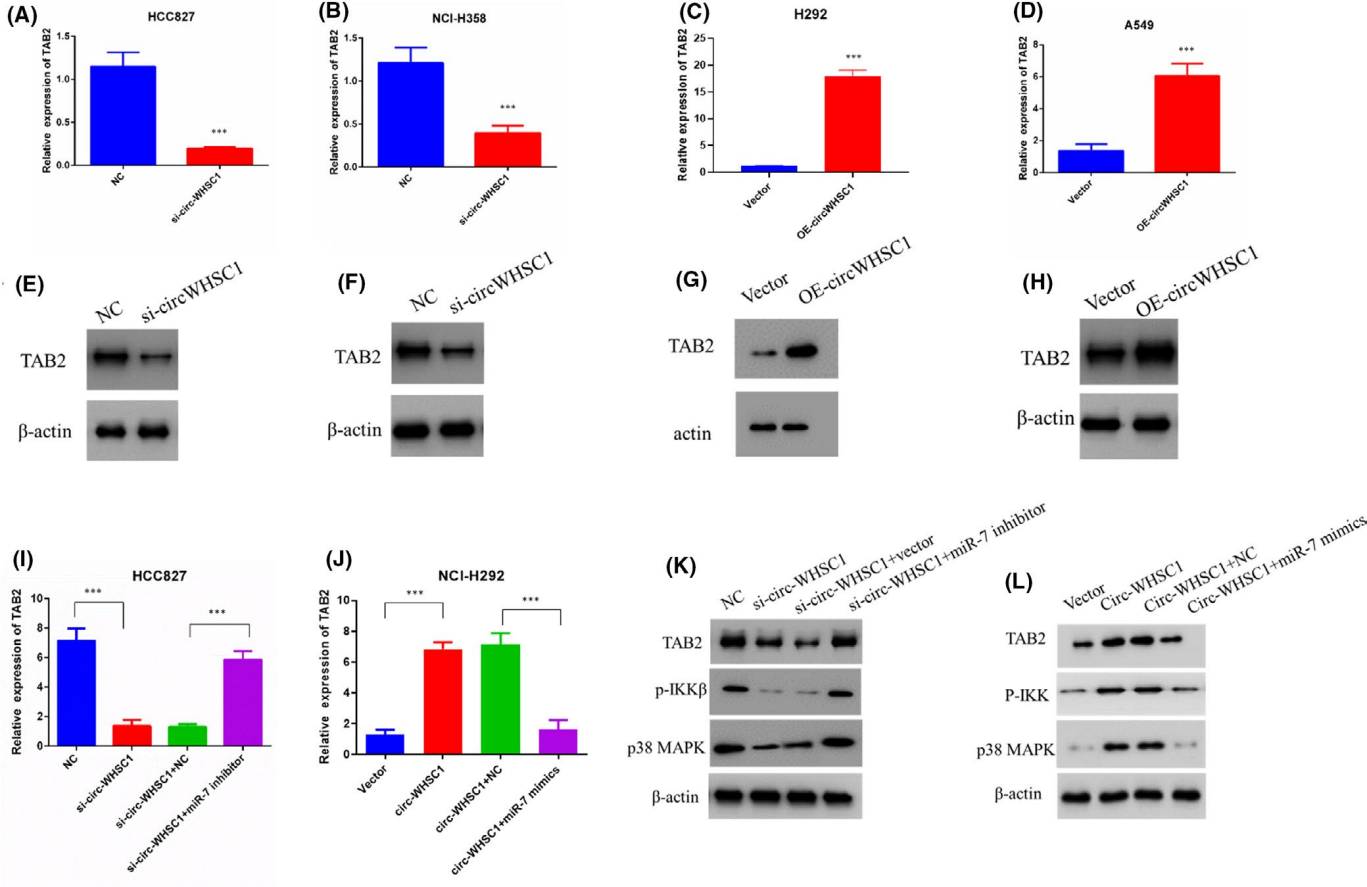
circ‐WHSC1 regulates TAB2 expression through miR‐7. (A) TAB2 mRNA in HCC827 NC and si‐circ‐WHSC1 cells. (B) TAB2 mRNA level in NCI‐H358 NC and si‐circ‐WHSC1 cells. (C) TAB2 mRNA level in NCI‐H292 Vector and OE‐circ‐WHSC1 cells. (D) TAB2 mRNA level in A549 Vector and OE‐circ‐WHSC1 cells. (E‐H) Protein levels of TAB2 in NSCLC cells. (I) miR‐7 inhibitor was transfected into HCC827 si‐circ‐WHSC1 cells. TAB2 mRNA levels were detected. (J) miR‐7 mimics were transfected into NCI‐H292 circ‐WHSC1 overexpression cells. TAB2 mRNA levels were detected. (K) The protein level of TAB2 and downstream proteins in HCC827 cells identified by Western blot. (L) The protein level of TAB2 and downstream proteins in NCI‐H292 cells identified by Western blot. NSCLC, non‐small cell lung cancer

We first detected the mRNA and protein level of TAB2 in circ‐WHSC1 overexpressed or circ‐WHSC1 silenced cells. Results showed that silencing of circ‐WHSC1 effectively impaired mRNA and protein levels of TAB2 (Figure 4A,B,E,F), and overexpression of circ‐WHSC1 also enhanced TAB2 mRNA and protein levels (Figure 4C,D,G,H). To figure out the function of miR‐7 in circ‐WHSC1‐induced TAB2 regulation, we detected TAB2 expression by transfecting miR‐7 inhibitor into circ‐WHSC1‐silenced cells. Data showed that the inhibition of miR‐7 rescued TAB2 mRNA levels (Figure 4I). The transfection of miR‐7 mimics effectively blocked TAB2 upregulation that induced by overexpression of circ‐WHSC1 (Figure 4J). The TAB2 adaptor protein connects TAK1 and TRAF6, thereby mediating TAK1 activation.^23^ Once activated, TAK1 phosphorylates the MAPK kinases MKK4 and MKK3/6, which activate JNK and p38 MAPK, respectively.^24^ TAK1 and TRAF6 also phosphorylate NF‐κB‐inducible kinase (NIK) to induce subsequent IKK activation, thereby activating the NF‐κB pathway.^24^, ^25^ p38 MAPK and IKKβ are two important downstream proteins of TAB2. We detected the activation of p38 MAPK and IKKβ by Western blot. The results showed that circ‐WHSC1 could promote TAB2 expression and enhance the activation of TAB2 downstream protein p38 MAPK and IKKβ (Figure 4K,L).

**FIGURE 5:**
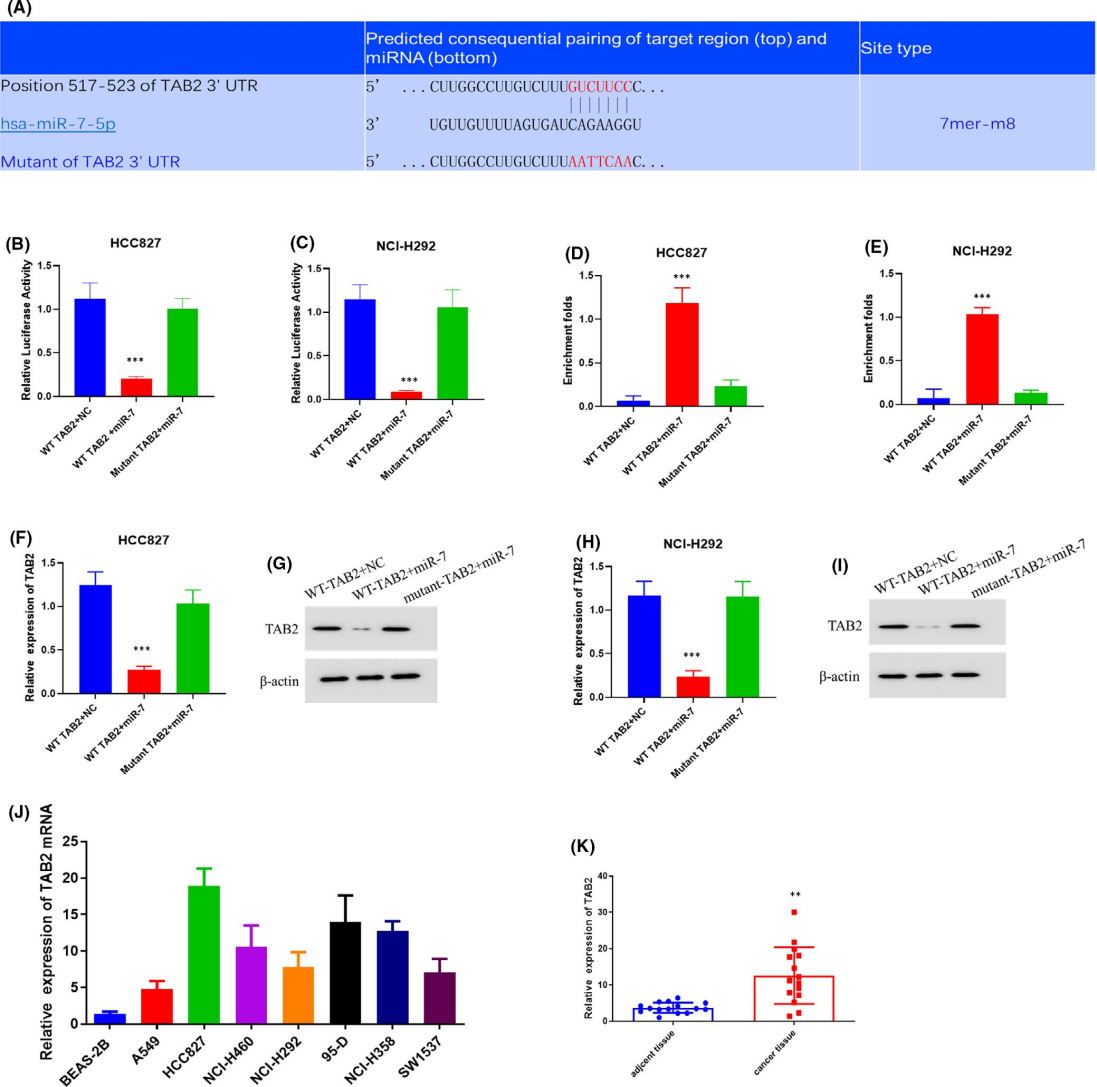
The binding site between miR‐7 and TAB2. (A) The predicted and mutant binding site between miR‐7 and TAB2. (B) The luciferase activity in HCC827. (C) The luciferase activity in NCI‐H292. (D) RNA pull down was carried out in HCC827. (E) RNA pull down was carried out in NCI‐H292. (F) mRNA level of TAB2 in HCC827. (G) Protein level of TAB2 in HCC827. (H) mRNA level of TAB2 in NCI‐H292. (I) Protein level of TAB2 in NCI‐H292. (J) mRNA level of TAB2 in lung cancer cells. (K) mRNA level of TAB2 in NSCLC tissue. NSCLC, non‐small cell lung cancer

We performed luciferase assay and RNA pull‐down assay to confirm the binding site between miR‐7 and TAB2 (Figure 5A‐E). The results showed that the miR‐7 could effectively decrease the luciferase activity of wild type of TAB2 3′UTR without influencing luciferase activity of mutant of TAB2 3′UTR (Figure 5B,C). The mutation of TAB2 3′UTR also blocked the binding between TAB2 and miR‐7 (Figure 5D,E). We also transfected the expression plasmids and miR‐7 into HCC827 and NCI‐H292 cells. qRT‐PCR and Western blot showed that the miR‐7 could not influence the expression of 3′UTR mutant TAB2 (Figure 5F‐I). All these results confirmed the binding site between miR‐7 and TAB2. These results strongly suggest that circ‐WHSC1 regulates TAB2 expression through miR‐7 in lung cancer cells.

### 3.6 circ‐WHSC1 promoted cancer progression through miR‐7/TAB2 axis

**FIGURE 6:**
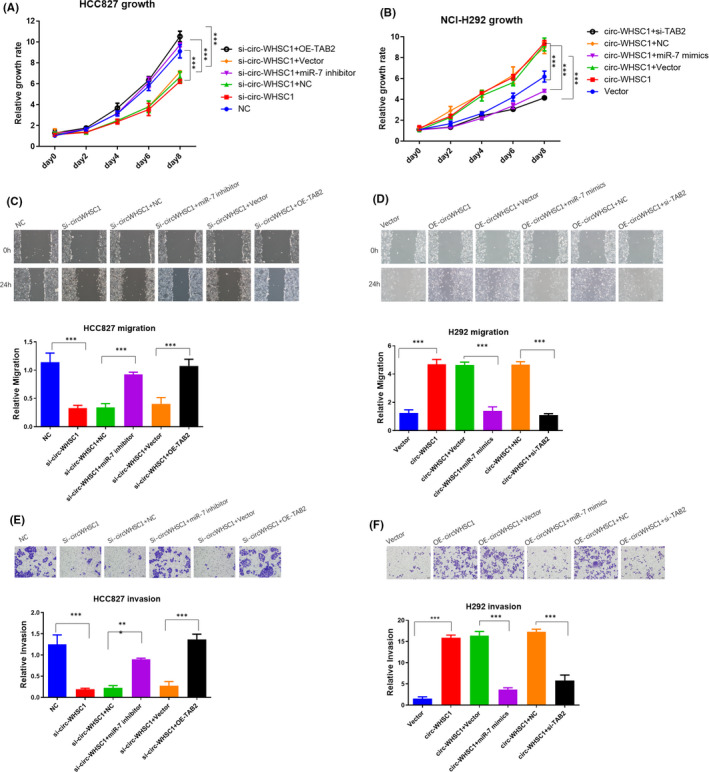
circ‐WHSC1 regulates lung cancer progression through miR‐7/TAB2 axis. (A) We transfected miR‐7 inhibitor or TAB2 overexpression plasmids into HCC827 si‐WHSC1 cells. Growth rates were detected. (B) We transfected miR‐7 mimics or TAB2 siRNA into NCI‐H292 WHSC1 overexpression cells. Growth rates were detected. (C) Migration was detected in HCC827. (D) Migration was detected in NCI‐H292. (E) Invasion was detected in HCC827. (F) Invasion was detected in NCI‐H292

We carried out the following experiments to investigate whether miR‐7/TAB2 axis was responsible for circ‐WHSC1‐induced cancer progression. We detected the growth rate by transfecting miR‐7 inhibitor or TAB2 overexpression plasmids into si‐circ‐WHSC1 HCC827 cells. Results showed that miR‐7 inhibition or TAB2 overexpression could rescue circ‐WHSC1‐silencing‐induced cell growth, migration and invasion inhibition (Figure 6A,C,E). Further, miR‐7 mimics or the silencing of TAB2 could effectively abrogate the tumour progression induced by circ‐WHSC1 overexpression (Figure 6B,D,F). These results showed that circ‐WHSC1 promoted NSCLC progression through the miR‐7/TAB2 axis.

### 3.7 Confirmation of circ‐WHSC1/miR‐7/TAB2 axis

**FIGURE 7:**
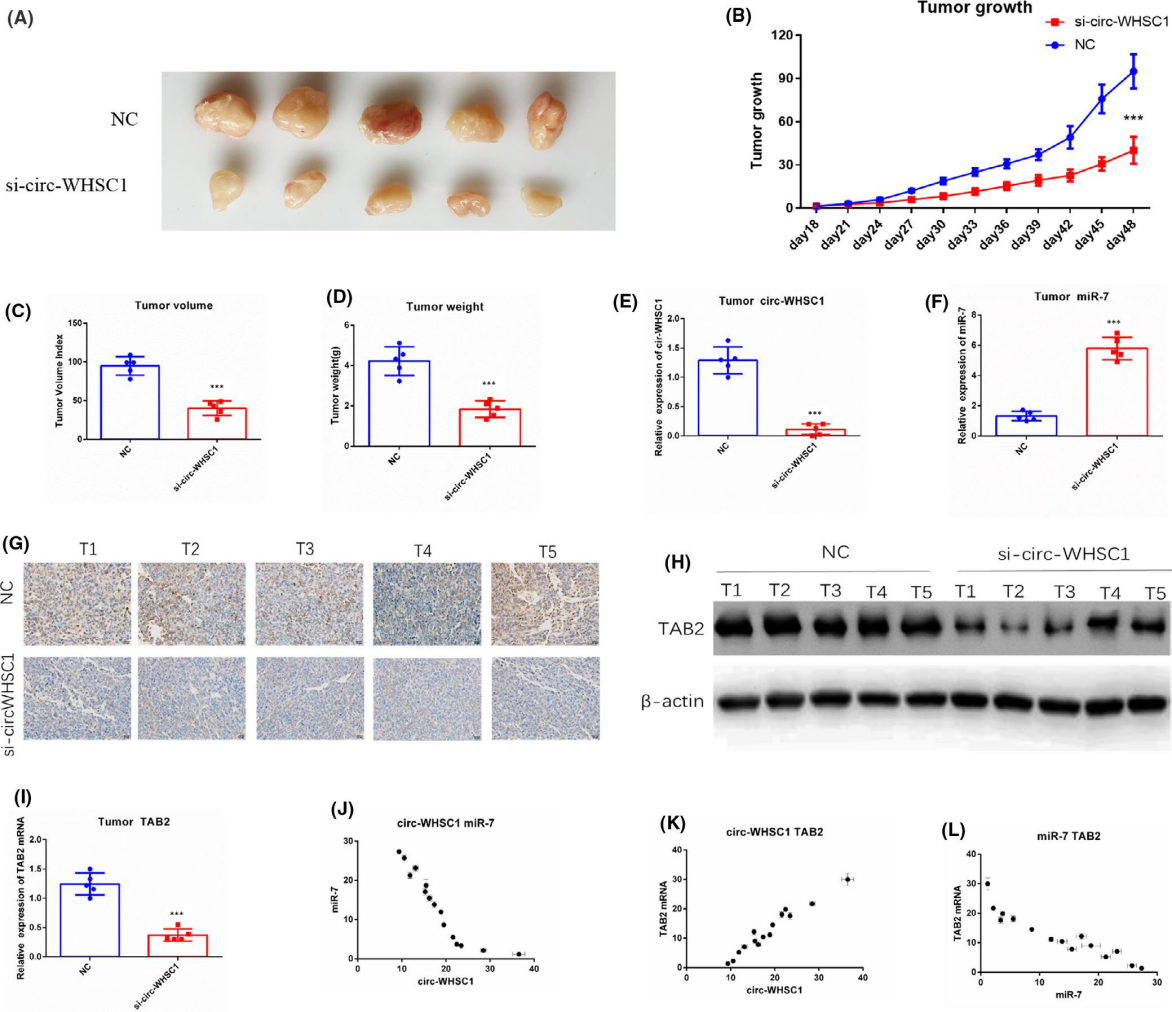
circ‐WHSC1/miR‐7/TAB2 exists in vivo. (A) 3 × 10^6^ HCC827 cells were transplanted into nude mice subcutaneously. 48 days after transplantation, tumours were collected. (B) The tumour growth rate was calculated as: (length × width^2^)/2. (C) The volume of tumour at 48 days after transplantation. (D) The weight of tumour at 48 days after transplantation. (E) Levels of circ‐WHSC1 in tumours. (F) Levels of miR‐7 in tumours. (G) TAB2 protein levels were detected in tumours by immunohistochemistry. (H) TAB2 protein levels were detected in tumours by Western blot. (I) The TAB2 protein level was analysed by qRT‐PCR. (J) circ‐WHSC1 expression was negatively correlated with miR‐7 expression in lung cancer tissues. (K) circ‐WHSC1 expression was positively correlated with TAB2 mRNA expression in NSCLC tissue. (L) miR‐7 expression was negatively correlated with TAB2 mRNA expression in NSCLC tissues. NSCLC, non‐small cell lung cancer

Further, we detected the functions of circ‐WHSC1/miR‐7/TAB2 in vivo by performing experiments in nude mice. None sense control (NC) and si‐circ‐WHSC1 HCC827 cells were inoculated subcutaneously into nude mice. We recorded length and width of the tumours every 3 days. As shown by the data, silencing of si‐circ‐WHSC1 effectively impaired the growth rate of HCC827 in comparison with NC group (Figure 7A,B). Silencing of circ‐WHSC1 also reduced the tumour volume and tumour weight (Figure 7C,D).

We also performed qRT‐PCR and Western blot to identify the expressions of circ‐WHSC1, miR‐7 and TAB2 in NSCLC tissues. Data showed that miR‐7 was significantly upregulated in si‐circ‐WHSC1 HCC827 cells (Figure 7E,F). The silencing of circ‐WHSC1 also effectively reduced both the mRNA and protein expression of TAB2 (Figure 7G,H,I). All results indicated that tumours with a higher expression of circ‐WHSC1 (Figure 7E) had a relatively lower level of miR‐7 (Figure 7F) and higher mRNA and protein levels of TAB2 (Figure 7G,H,I). All the results proved the existence of circ‐WHSC1/miR‐7/TAB2 pathway in vivo.

We also investigated the relationship between circ‐WHSC1, miR‐7 and TAB2 in lung cancer tissues. The data showed that circ‐WHSC1 and miR‐7 expression were negatively correlated, and circ‐WHSC1 and TAB2 expression were positively correlated (Figure 7J,K). Moreover, miR‐7 was negatively correlated with the expression of TAB2 (Figure 7L). All the results collectively confirmed circ‐WHSC1/miR‐7/TAB2 axis.

## 4 DISCUSSION

In this research, we discovered that circ‐WHSC1 was highly expressed in NSCLC cells and tissues. circ‐WHSC1 enhanced the tumour growth, migration and invasion of NSCLC cells. Second, the data proved that overexpression of circ‐WHSC1 could abrogate the suppressive influence of miR‐7 and promote malignant progression in lung cancer. We also found the binding site between circ‐WHSC1 and miR‐7, which provided more information for future studies. Third, we discovered that circ‐WHSC1 upregulated TAB2 expression via sponging miR‐7. Inhibition of the circ‐WHSC1/miR‐7/TAB2 pathway could effectively block the progression of lung cancer. In summary, our research proved the existence and functions of circ‐WHSC1 in lung cancer, for the first time.

Circular RNAs are newly found forms of RNA and are widely expressed in mammalian cells. Circular RNAs have a higher expression than their linear isomers.^26^ Stability and gene conservation are the two most important features of circRNA.^27^ All these features suggest that circular RNAs are more promising as cancer biomarkers compared with other ncRNAs, such as micro‐RNAs and lnRNAs.^28^ Recently, the function of circular RNA in cancer has drawn increasing attention from researchers. Uncovering the function of circular RNA will provide more new ideas for cancer therapy.

Lung cancer has been a heavy global burden for worldwide health. During the last decades, the diagnosis and therapy of NSCLC have developed slowly. Five‐year survival rate of advanced‐stage patient is under 20%.^29^, ^30^ Most patients with locally advanced or metastatic disease lost the opportunity for operation. The investigation of NSCLC early diagnostic and oncogenic factors is conducive to new target therapies.

Non‐small cell lung cancer progression is a complex process involving various factors. Existing studies have reported the circRNA expression profiles in NSCLC tissues; however, the specific function of circRNAs has not been elucidated clearly until now. Therefore, research on circRNAs in NSCLC is of great importance for NSCLC treatment.

circ‐WHSC1 is generated from the *WHSC1* gene. *WHSC1* is reported to play carcinogenic roles in cancers^31^; however, circ‐WHSC1 has not been explored until now. In this research, we found the existence of circ‐WHSC1 in NSCLC for the first time and revealed its functional role.

A significant upregulation of circ‐WHSC1 was detected in both NSCLC cells and tissues (Figure 1). circ‐WHSC1 could promote the proliferation, migration and invasion of cancer cells, and loss‐of‐function experiments demonstrated that the silencing of circ‐WHSC1 impaired the progression of lung cancer (Figure 2). Furthermore, animal experiments proved that the silencing of circ‐WHSC1 inhibited tumour proliferation in vivo (Figure 7A,B). All the results encouraged us to explore the oncogenic mechanisms of circ‐WHSC1 in NSCLC.

One of the most important functions of circRNA is to regulate gene expression by sponging micro‐RNAs.^32^ Micro‐RNAs are an important member of ncRNAs which is critical to cellular activities. It is known that the mRNAs transcription could be abrogated by the binding of micro‐RNA.^18^ We focussed on miR‐7 based on bioinformatical analysis. It has been reported that miR‐7 could regulate numerous oncogenes, and inhibition of miR‐7 would advance the mechanism of cancer progression.^33^ miR‐7 regulates synuclein to influence the progression of Parkinson's disease.^34^ In addition, miR‐7 inhibition activated the mTOR pathway and promoted cell proliferation.^35^, ^36^ In addition, miR‐7 could target numerous oncogenic genes in cancer‐associated pathways, such as *EGFR*,^37^ *PIK3CD*,^38^ *Ack1* ^39^ and *Raf1*,^40^ indicating the significant suppressive roles in cancers. We performed qRT‐PCR, luciferase reporter assays and biotin pull‐down assays and confirmed that circ‐WHSC1 acted as a sponge for miR‐7.

Results also exhibited that miR‐7 was expressed at low levels in NSCLC cells and tissues, which was consistent with the expression of circ‐WHSC1 (Figure 3). The CCK8, migration and invasion assays also confirmed that miR‐7 was responsible for circ‐WHSC1‐induced cancer progression (Figure 6).

Further, we searched for the miR‐7 target gene using bioinformatics analysis. TAB2 caught our attention. TAB2 is a protein that is an activator of MAP3K7/TAK1. TAB2 could form a kinase complex with TAB1, which links TRAF6 and MAP3K7. The complex regulates signal transduction by activating NF‐kappaB, which influences osteoclasts development.^41^ TAB2 has been reported to contribute to steroid antagonist resistance in prostate and breast cancer.^42^, ^43^, ^44^

In addition, TAB2 also displays a regulatory function in transcriptional gene expression, in conjunction with NCoR.^45^ Several reports have shown that TAB2 functions as ‘molecular beacon’ to integrate the transcriptional response that are critical for disease progression.^46^ In our study, we found that circ‐WHSC1 could upregulate TAB2 expression through miR‐7. Furthermore, TAB2 was responsible for the cancer progression induced by circ‐WHSC1. Till now, most of the researches mainly focussed on the functions of TAB2, but few researches reported the factors regulating TAB2 expression. In this research, we find a novel pathway that regulates TAB2 expression, which provides information for further research on TAB2.

In conclusion, our research provides comprehensive evidence that circ‐WHSC1 is an oncogenic factor in NSCLC progression. Results showed that upregulation of circ‐WHSC1 enhanced lung cancer progression by impairing the level of miR‐7 and increasing TAB2 expression. Furthermore, inhibition of the circ‐WHSC1/miR‐7/TAB2 axis could effectively block the progression of NSCLC. circ‐WHSC1/miR‐7/TAB2 axis might be a potential therapeutic target for NSCLC.

## CONFLICTS OF INTEREST

All authors declared no potential conflicts of interest.

## AUTHOR CONTRIBUTIONS

**Sisi Guan:** Project administration (lead); Resources (lead); Validation (lead). **Li Li:** Data curation (lead); Formal analysis (lead); Project administration (lead). **Wen‐Shu Chen:** Conceptualization (equal); Data curation (equal); Formal analysis (equal). **Wen Yang Jiang:** Investigation (equal); Methodology (equal). **Yun Ding:** Methodology (equal); Project administration (equal). **Li‐Lan Zhao:** Project administration (equal). **Yi‐Fan Shi:** Resources (equal); Software (equal). **Jun Wang:** Validation (equal); Visualization (equal). **Qi Gui:** Funding acquisition (equal); Writing‐original draft (equal); Writing‐review & editing (equal). **Cheng‐Cheng Xu:** Project administration (equal); Writing‐original draft (equal). **Yang Cheng:** Investigation (lead); Methodology (lead); Resources (lead). **Wenjuan Zhang:** Funding acquisition (lead); Supervision (lead).

## STATEMENT OF ANIMAL RIGHTS

All animal experiments were under the direction of The Central Hospital of Wuhan.

## Supporting information

Supplementary Material

## Data Availability

The data that support the findings are available from the corresponding author upon reasonable request.
