# Supplementary figures and images for: Circular RNA WHSC1 exerts oncogenic properties by regulating miR‐7/TAB2 in lung cancer
Source: J Cell Mol Med. 2021 Sep 22;25(20):9784–95. doi: 10.1111/jcmm.16925 (PMC8505844; doi:10.1111/jcmm.16925)

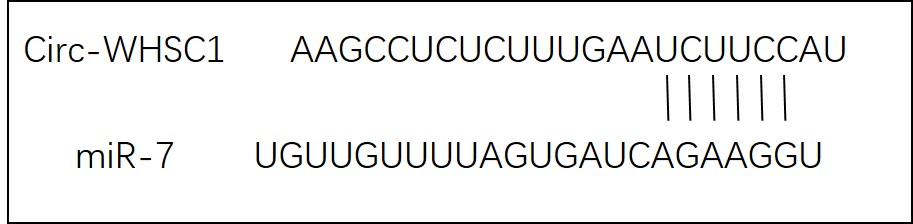


Supplementary Figure 1

The binding site between circ-WHSC1 and miR-7.

Supplement: Supplementary file 1 — Supplementary Material [file JCMM-25-9784-s001.docx]
